# Cry1Ac and Vip3Aa proteins from *Bacillus thuringiensis* targeting Cry toxin resistance in *Diatraea flavipennella* and *Elasmopalpus lignosellus* from sugarcane

**DOI:** 10.7717/peerj.2866

**Published:** 2017-01-17

**Authors:** Ana Rita Nunes Lemes, Camila Soares Figueiredo, Isis Sebastião, Liliane Marques da Silva, Rebeka da Costa Alves, Herbert Álvaro Abreu de Siqueira, Manoel Victor Franco Lemos, Odair Aparecido Fernandes, Janete Apparecida Desidério

**Affiliations:** 1 Department of Biologia Aplicada á Agropecuária, Universidade Estadual Paulista, Jaboticabal, São Paulo, Brazil; 2 Department of Agronomia, Universidade Federal Rural de Pernambuco, Recife, Pernambuco, Brazil; 3 Departamento de Fitossanidade/FCAV/UNESP, Universidade Estadual Paulista, Jaboticabal, São Paulo, Brazil

**Keywords:** Biological control, Insecticidal proteins, Competition assay

## Abstract

The biological potential of Vip and Cry proteins from *Bacillus* is well known and widely established. Thus, it is important to look for new genes showing different modes of action, selecting those with differentiated entomotoxic activity against *Diatraea flavipennella* and *Elasmopalpus lignosellus*, which are secondary pests of sugarcane. Therefore, Cry1 and Vip3 proteins were expressed in *Escherichia coli*, and their toxicities were evaluated based on bioassays using neonate larvae. Of those, the most toxic were Cry1Ac and Vip3Aa considering the LC_50_ values. Toxins from *E. coli* were purified, solubilized, trypsinized, and biotinylated. Brush Border Membrane Vesicles (BBMVs) were prepared from intestines of the two species to perform homologous and heterologous competition assays. The binding assays demonstrated interactions between Cry1Aa, Cry1Ac, and Vip3Aa toxins and proteins from the BBMV of *D. flavipennella* and *E. lignosellus*. Homologous competition assays demonstrated that binding to one of the BBMV proteins was specific for each toxin. Heterologous competition assays indicated that Vip3Aa was unable to compete for Cry1Ac toxin binding. Our results suggest that Cry1Ac and Vip3Aa may have potential in future production of transgenic sugarcane for control of *D. flavipennella* and *E. lignosellus*, but more research is needed on the potential antagonism or synergism of the toxins in these pests.

## Introduction

Sugarcane is grown in several countries of tropical and sub-tropical regions, playing an important role in the economy, especially sugar and alcohol production (Moore, 2005; Srikanth, Subramonian & Premachandran, 2011). This crop is attacked by several pests, and in Brazil such losses may be as high as 10% due to the sugarcane borer, *Diatraea saccharalis* (Fabricius, 1794) and spittlebugs, *Mahanarva* spp. (Oliveira et al., 2014).

Moreover, some secondary pests, such as another species of the sugarcane borer, *Diatraea flavipennella* (Box, 1931) and the lesser cornstalk borer, *Elasmopalpus lignosellus* (Zeller, 1848) may also be problematic. The larvae of *D. flavipennella* can cause direct or indirect losses similar to *D. saccharalis*. Direct losses are due to galleries burrowed by larvae in sugarcane stems, which can lead to yield reductions or ‘dead heart’ (apical bud death), particularly in 3-month-old plants (Pinto, Machado & Oliveira, 2009; Garcia, 2013). This species is abundant in sugarcane producing areas of Northeastern Brazil and is considered more damaging than *D. saccharalis* (Mendonça et al., 1996; Pinto, 2006). Conversely, *E. lignosellus* is a polyphagous insect whose larvae destroy the meristematic tissue bellow the soil surface and/ or the phloem vessels of saplings, affecting crop stand (Viana, Cruz & Waquil, 2000; Viana, 2004).

For lepidopteran pest control, *Bacillus thuringiensis* (Berliner, 1915) is the most recommended strategy for biopesticdes. Transgenic crops expressing *B. thuringiensis* insecticidal proteins have been used to control such pests worldwide since 1996 (Tabashnik, Brevault & Carrière, 2013; James, 2014). These genetically modified crops are less dependent on chemical sprays, and are more cost-effective and target-specific, causing less environmental impact and human health risks (James, 2014). However, there are no sugarcane varieties expressing insecticidal proteins to control sugarcane pests.

One of the concerns about widespread use of Bt plants is that, under selection pressure, increases in the emergence of Bt-resistant insects will occur, as well as the onset of secondary pests with decreased susceptibility to the transgenic toxin attacking transgenic crops (Zhang et al., 2013). The evolution of pest resistance can reduce economic and environmental benefits of the transgenic crops. A few field resistance cases have been reported involving transgenics incorporating Cry1 toxins, including *Busseola fusca* (Fuller) in South African maize crops (Van Rensburg, 2007), *Helicoverpa armigera* (Hübner) in Chinese cotton (Liu et al., 2010), *Spodoptera frugiperda* (Walker) in Puerto Rican maize (Storer et al., 2010), *Pectinophora gossypiella* (Saunders) in Indian cotton (Dhurua & Gujar, 2011) and *Diabrotica virgifera* (LeConte) in North American maize (Gassmann et al., 2011); and recently, has also been detected in Brazilian maize crops infested by *S. frugiperda* (Farias et al., 2014).

Commercial varieties of pyramided crops are being released to circumvent targeted-pest resistance and broaden the spectrum of target insects. Pyramided plants contain more than one Bt *cry* gene; however, the effect on secondary pests has been less studied. Some sugarcane varieties have already been developed with Bt *cry* genes, such as *cry1Ab* and *cry1Ac*, to control primary pests, mainly *D. saccharalis* (Weng et al., 2011).

In the present investigation, we evaluated the effect of some Cry1 and Vip3 proteins on *D. flavipennella* and *E. lignosellus*, and to evaluated binding of both Cry1Aa, Cry1Ac, and Vip3Aa to proteins in insect midgut cells, reflected in a binding model of these toxins for these pests. The results suggest the most promising gene combination for genetically modified sugarcane with pyramided Bt toxins targeting secondary pests.

## Material and Methods

### Insects

*D. flavipennella* were kindly provided by the Insect Pathology Laboratory of the Federal Rural University of Pernambuco (Universidade Federal Rural de Pernambuco—UFRPE), in Recife, PE, Brazil. *E. lignosellus* insects were purchased from BUG Biological Agents, in Piracicaba, SP, Brazil.

### Cry1 and Vip3 protein expression

**Figure 1:**
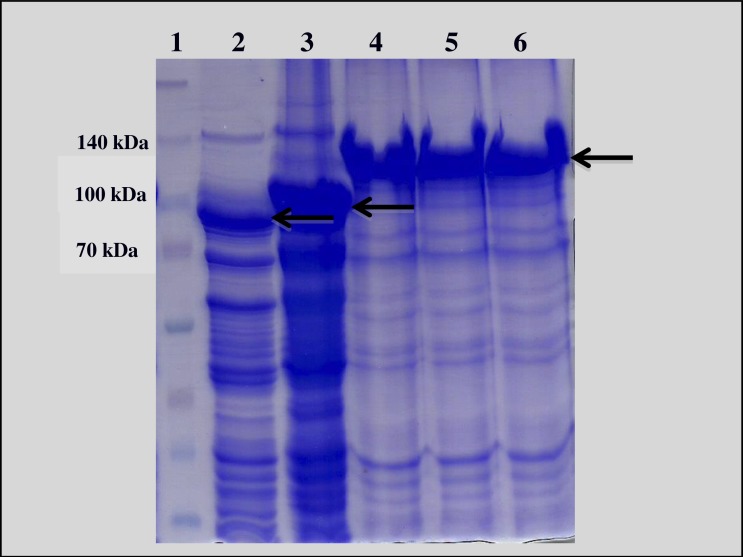
SDS_PAGE 12% of recombinant Cry1 and Vip3 proteins. **1**—Molecular mass marker “Spectra™ Multicolor Broad Range Protein Ladder” (Fermentas); **2**—Vip3Ca; **3**—Vip3Aa; **4**—Cry1Aa; **5**—Cry1Ac; **6**—Cry1Ca. The arrows indicate the protoxins.

*Escherichia coli* strain XL-Blue samples harboring *B. thuringiensis cry*1 genes (*cry1Aa, cry1Ac*, and *cry1Ca*) were provided by Ruud de Maagd (Plant Research International, Wageningen, Netherlands). The Cry1 proteins were produced according to Herrero et al. (2004) and were analyzed by 12% SDS-PAGE (Fig. 1).

The construct containing *vip3Aa* gene in *E. coli* was previously developed in our laboratory by isolation of the gene encoding the protein from *B. thuringiensis* HD-125, according to Mendes (2011), and following standard cloning procedures into the pETSumo plasmid vector (Invitrogen). Vip protein expression was carried out as described by Chakroun et al. (2012), after final induction using isopropyl *β*-D-thiogalactoside (IPTG) at 1 mM and, then analyzed by 12% SDS-PAGE (Fig. 1). The quantification of expressed protein concentration to be used in the bioassays was made by optical densitometry in SDS-PAGE gels using the software ImageQuant TL 8.1 (GE Healthcare Bio-Sciences AB, Uppsala, Sweden), using BSA (bovine serum albumin) as the standard. The Vip3Ca protein from *B. thuringiensis* was supplied by Professor Juan Ferré (University of València, Campus in Burjassot, Spain).

Analysis of cell lysates revealed a protein band with the expected molecular weighs for Cry1 (135 kDa), Vip3Ca (85 kDa) and Vip3Aa (100 kDa), slightly larger since the expressed protein was an additional 15 kDa due the fused SUMO protein.

### Bioassays

Bioassays were carried out to evaluate the biocidal activity of *B. thuringiensis* proteins on *D. flavipennella* and *E. lignosellus* neonate larvae, which were fed artificial diet specifically designed by Araújo et al. (1985) and Viana (1993), respectively. Several concentrations of Cry1Aa, Cry1Ac, Cry1Ca, Vip3Aa and Vip3Ca were evaluated, testing each one on 16 neonate larvae in culture plates (Cell Wells; Corning Glass Works, Corning, New York) with three replicates, a total of 48 larvae per concentration.

The plates were kept at 25 °C ±2 °C, 70 ± 10% RH and 14:10 LD photoperiod. Mortality was recorded after 15 and 7 days for *D. flavipennella* and *E. lignosellus*, respectively. As control, we used distilled water, protein solubilization buffer and *E. coli* cell lysates (without host vector expression) with *cry* and *vip3* genes, as performed by Crialesi-Legori et al. (2014). LC_50_ determination was by mortality counts for each protein and evaluated concentrations with Probit analysis (Finney, 1971) using Polo-Plus software (LeOra Software, Berkeley, CA, USA).

### Protein purification, activation and labelling

The Cry1 proteins were purified using the HiTrap HP ion-exchange column (Amershan Biosciences), and the Vip3Aa was purified by affinity histidine using HisTrap HP column (Amershan Biosciences). Cry1 proteins were activated prior to purification, while Vip3 ones were first purified, and then trypsinized, due to the presence of a histidine tail required for the HisTrap column during purification steps. The protoxins were trypsinized with 10% bovine trypsin (Sigma) at 37 °C for 1h and 30 min, shaken at 200 rpm, and enzyme inactivation by centrifugation at 17,000 × g at 4 °C for 10 min.

Proteins were biotin-labelled using the ECL Biotinylation kit (GE Healthcare). The labelling reaction was carried out for 2 h using 1,000 µg of each protein plus 40-µl biotinylation reactive, under slight shaking and at 25 °C. Proteins were eluted from the G-25 column (P10Desalting; GE Healthcare, Munich, Germany) with 20 ml of phosphate buffered saline (PBS, pH 7.4), quantified by the Bradford method (Bradford, 1996) and kept at 4 °C.

### Brush-Border Membrane Vesicles preparation—BBMV

Midgut samples of fourth-instar larvae of *D. flavipennella* and *E. lignosellus* were collected, washed, pooled, and stored in ice-cold MET buffer (250 mM mannitol, 17 mM Tris–HCl and 5 mM EGTA; pH 7.5), frozen in liquid nitrogen and kept at −80 °C (Hernández, Rodrigo & Ferré, 2004). This procedure followed the differential magnesium precipitation method described by Wolfersberger et al. (1987) and quantified by the Bradford method (1996).

### Ligand-Blotting procedure

Ligand blotting was carried out according to the description by Abdelkefi-Mesrati et al. (2011), using 40-µg midgut sample and 40 nM of each toxin. After electrophoresis, the proteins were transferred to a PVDF membrane (GE-Healthcare). which was then subjected to blocking using 10 ml of Tris buffered saline (TBS) (50 mM Tris-Cl, pH 7.5 containing 150 mM NaCl) including 5% powdered skim milk and 0.1% Tween-20, with occasional shaking during 1 h. The membrane was washed with TBST (Tris buffered saline 1X containing 0.1% Tween-20) for 5 min and afterward incubated for 2 h with each biotin-labeled toxin (40nM), also diluted in TBST. Subsequently, the membranes were washed again with TBST three times, 5 min each. Blots were incubated 1 h in streptavidin/peroxidase conjugate (HRP) (GE Healthcare), for specific ligation to biotin. Finally, the membranes were washed three times with TBST and developed using 10 ml of NBT/BCIP solution (Roche Diagnostics).

### Homologous competition assays among Cry1Aa, Cry1Ac and Vip3Aa and heterologous between Cry1 and Vip3

The homologous and heterologous competition assays were performed in accordance with procedures reported by Abdelkefi-Mesrati et al. (2011). Biotin-labeled toxin (100 ng) were incubated with an excess of unlabeled competing toxin (50, 100, 500 and 1,000×) in the presence of 10 µg of BBMVs of each pest insect for 1 h and 30 min at 28 °C with 140 rpm shaking. To stop the reaction, samples were centrifuged at 13.000 rpm at 4 °C for 10 min. BBMVs were washed twice with 500 µl PBS, centrifuge similarly each time, and finally dissolved in 20 µl PBS. The samples were later incubated with 5 ml of Laemmli 4X sample buffer at 100 °C for 5 min and subjected to electrophoresis in 9% SDS-PAGE gels. After the electrophoresis, the samples were transferred to PVDF membranes (GE Healthcare). The biotinylated proteins bound to the BBMVs were detected using the streptavidin/peroxidase conjugate diluted at 1:2,000 (HRP) (GE Healthcare) and developed in 10 ml of NBT/BCIP (Sigma) solution.

## Results

### Bioassays

The toxicity of Cry1Aa, Cry1Ac, Cry1Ca, Vip3Aa and Vip3Ca were evaluated using neonate larvae of *D. flavipennella* and *E. lignosellus,* and the lethal concentrations for 50% mortality (LC_50_) were compared (Table 1). Cry1Ac was most toxic to *D. flavipennella* and *E. lignosellus* (LC_50_ = 8.6 and 15.6 ng/cm^2^, respectively) of all protoxins tested. There were overlapping confidence intervals for Cry1Aa and Cry1Ca in both insects, suggesting a similar intermediate toxicity response. While Vip3Aa was similar to Cry1Aa and Cry1Ca in toxicity to *E. lignosellus*, it was much less toxic to *D. flavipennella* (LC_50_ = 495ng∕cm^2^). Vip3Ca was not toxic to either insect.

**Table 1:** Susceptibility of * Diatraea flavipennella* and *Elasmopalpus lignosellus* neonate larvae to proteins Cry1 and Vip3.

Insect	Protein	LC_50_ (CI min–max)^b^	*b* ± SE^a^	Chi-square
*D. flavipennella*	**Cry1Aa**	106 (58.2–181)^*^	1.31 ± 0.17	6.72
	**Cry1Ac**	8.60 (3.40–13.5)	1.85 ± 0.26	8.04
	**Cry1Ca**	64.0 (38.5–102)^*^	0.84 ± 0.13	2.70
	**Vip3Aa**	495 (329–1,150)	1.66 ± 0.51	1.08
	**Vip3Ca**	>2,000	ND^c^	ND
*E. lignosellus*	**Cry1Aa**	73.6 (40.8–131)^**^	0.50 ± 0.41	16.94
	**Cry1Ac**	15.6 (9.30–24.6)	0.76 ± 0.53	19.02
	**Cry1Ca**	36.1 (20.9–61.3)^**^	0.68 ± 0.54	17.56
	**Vip3Aa**	49.9 (28.7–84.2)^**^	0.65 ± 0.46	21.61
	**Vip3Ca**	481 (315–772)	0. 57 ± 0.44	8.11

**Notes.**

The values represent the mean of three replicates with 16 larvae per replicate (*n* = 48).

a Line angular coefficient and Standard error.

b Values expressed in ng/cm^2^ with confidence interval (CI 95%).

c Undetermined.

* and ** non significative difference among LC_50_.

### Ligand blotting

**Figure 2:**
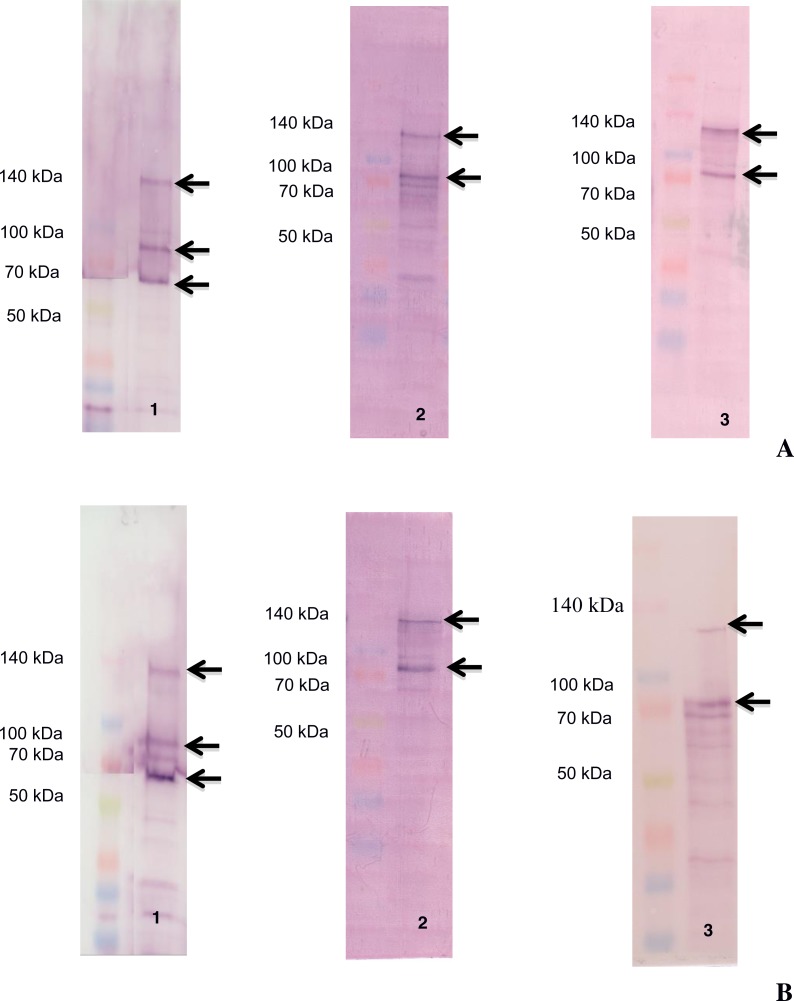
Ligand blot analysis of the binding of Cry1Aa (1), Cry1Ac (2) and Vip3Aa (3) toxin to BBMV proteins from * D. flavipennella* (A) and * E. lignosellus* (B). BBMVs proteins (40 µg) from each insect were transferred to PVDF membrane and incubated with 40 nmol of biotinylated toxins. Arrows indicate main BBMV proteins recognized by each toxin in each BBMV sample.

The ligand-blotting analysis demonstrated the binding of biotin labelled toxins (Cry1Aa, Cry1Ac and Vip3Aa) to proteins in the BBMVs of *D. flavipennella* an *E. lignosellus* (Fig. 2). Cry1Aa bound to proteins of molecular mass 65, 90, and 130 kDa in BBMV from both insects (Fig. 2, left panel). Cry1Ac bound to proteins of molecular mass 90 and 120 kDa in BBMV from both insects (Fig. 2, center panel). Vip3Aa was similar to Cry1Ac in binding 90 and 120 kDa proteins in *D. flavipennella* BBMV, but bound to a doublet around 90 kDa from *E. lignosellus* (Fig. 2, right panel).

### Homologous and heterologous competition assays between Cry1 and Vip3

**Figure 3:**
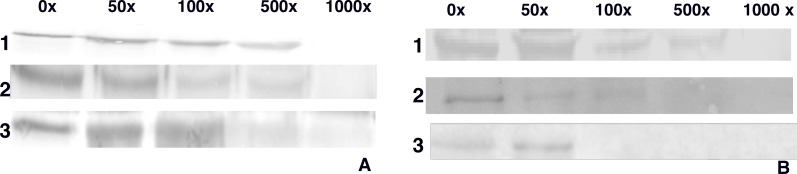
Homologous competition involving: (1) Cry1Aa, (2) Cry1Ac, (3) Vip3Aa biotin labeled, with excess of 50×, 100×, 500×, 1,000× of their respective unlabeled competing and 10 µg of BBMV from *D. flavipennella* (A) and *E. lignosellus* (B).

Homologous assays were carried out using Cry1Aa, Cry1Ac and Vip3Aa purified and activated proteins with BBMVs from *D. flavipennella* and *E. lignosellus* larvae. Homologous competition was observed with all three toxins incubated with a protein in BBMV from both insects, with varying amounts of unlabeled toxin required for competition (Fig. 3). With *D. flavipennella*, Cry1Aa and Cry1Ac were competed with 1000x unlabled toxin, whereas Vip3Aa was effectively competed with 500x unlabeled toxin (Fig. 3A), suggesting that it has a lower binding affinity. Similar competition of Cry1Aa and Cry1Ac was observed in *E. lignosellus*, but Vip3Aa was competed by only 100x unlabeled Vip3Aa (Fig. 3B).

**Figure 4:**
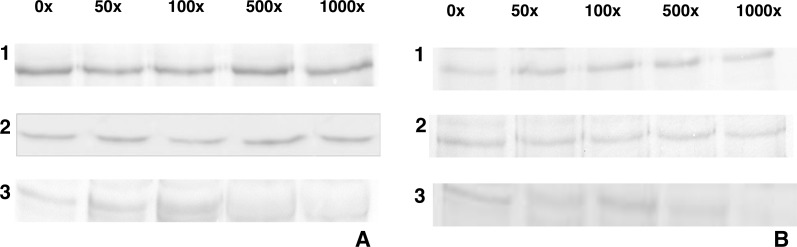
Heterologous competition. (1) Cry1Aa biotin-labeled and Vip3Aa unlabeled, (2) Cry1Ac biotin-labeled and Vip3Aa unlabeled, (3) Cry1Aa biotin-labeled and Cry1Ac unlabeled. Each reaction were prepared using excess of 50×, 100×, 500×, 1,000× from Vip3Aa unlabeled and Cry1Ac unlabeled and with 10 µg de BBMV from *D. flavipennella* (A) *E. lignosellus*(B).

For heterologous competition assays, excess Vip3Aa up to 10000x was unable to compete the binding of Cry1Aa or Cry1Ac to BBMV from either insect (Fig. 4), suggesting that this was a unique binding protein for Cry1A toxins. However, 500x unlabeled Cry1Aa was able to compete binding of Cry1Ac in both insects, which suggested that they share a common binding site in both insects.

## Discussion

Of the toxins evaluated in this study, Cry1Ac was most toxic to *D. flavipennella* and *E. lignosellus*, with the lowest LC_50_ and nonoverlapping confidence intervals with all other toxins (except with Cry1Ca in *E. lignosellus*). Therefore, both insects should be effectively controlled by Cry1Ac toxin. These results are similar to those published by Li & Bouwer (2014), who also observed the lowest LC_50_ for Cry1Ac compared to Cry1Aa and Cry1Ca in larvae of *Helicoverpa armigera* (Hübner, 1805) (Lepidoptera: Noctuidae).

However, to circumvent the development of resistance to Cry1Ac, as has been observed in many laboratory and field pests, pyramiding with another toxin with a different mode of action and binding site would be desirable. Vip toxins may be such candidates. Baranek et al. (2015) analyzed the toxicity of Vip3 proteins (Vip3Aa58 and Vip3Aa59) and found efficacy in *S. exigua* (Hübner) (Lepidoptera: Noctuidae). More broadly, other studies have shown Vip proteins are efficient to control other species of lepidopteran pests (Crialesi-Legori et al., 2014; Lemes et al., 2014; Marucci et al., 2015). Recently, Vip3Aa has been introduced in prominent crops to increase protection against lepidopteran pests, and also as part of a basic strategy for resistance management as response to an emerging resistance to Cry proteins (Downes & Mahon, 2012; Tabashnik, Wu & Wu, 2012). Of the Vip toxins tested in this study, Vip3Aa was similar in toxicity to other Cry1A toxins to *E. lignosellus*, but was less toxic to *D. flavipennella*. Vip3Ca, however, was not toxic to either insect.

The first generation of Bt-transgenic crops contained a single Bt toxin gene; meanwhile, a second emerging generation contained multiple copies of Bt genes. Therefore, it is important to evaluate not only the effect of insecticide proteins but also their interactions, ensuring that the interactions between toxins and larval membrane receptors will occur without competition among transgenic gene products. Future studies in *D. flavipennella* and *E. lignosellus* with Cry1Ac and Vip3Aa are needed to confirm whether these toxins can be pyramided in transgenic sugarcane for effect control.

According to literature reports, alkaline phosphatase molecular mass ranges from 62 to 68 kDa (Jurat-Fuentes & Adang, 2001; Jurat-Fuentes & Adang, 2006; Fernández et al., 2006) and aminopeptidase-N between 130 and 150 kDa (Rajagopal et al., 2003; Hua, Jurat-Fuentes & Adang, 2004; Pacheco et al., 2009), acting as a 130 kDa homodimer formed by 65 kDa monomers (Malik, Riazuddin & Riazuddin, 2006). We found Cry1 and Vip3 toxin binding proteins in both insects with similar molecular masses, although more work is needed to determine if these are the same proteins.

Besides these proteins, cadherin receptors with molecular masses of roughly 210 kDa are among the most high affinity binding proteins for Cry toxins (Flannagan et al., 2005; Hua, Jurat-Fuentes & Adang, 2004; Jurat-Fuentes & Adang, 2006). However, our binding blots were unable to determine if similar Bt receptors are found in *D. flavipennella* and *E. lignosellus.*

Nevertheless, it was found that some of the putative receptors detected in *D. flavipennella* and *E. lignosellus* for Cry1Aa, Cry1Ac and Vip3Aa could be similar based on estimates of molecular masses in gels. However, there may be some differences in Vip3Aa toxin binding proteins in BBMV from *E. lignosellus.*

Similar to our results, Han et al. (2014) also observed two or more bands in ligand-blotting assays results for BBMVs of *Chilo suppressalis* (Walker, 1635) (Lepidoptera: Pyralidae) and of *Sesamia inferens* (Walker, 1956) (Lepidoptera: Noctuidae). Moreover, these authors detected a strong binding affinity for Cry1Ac to *C. suppressalis* BBMVs compared to *S. inferens*. Cry1Ab and Cry1Ac also bound to BBMV from *S. frugiperda* and *D. saccharalis* Rang et al. (2004), but Macedo et al. (2012) found that Cry1Aa also bound to BBMV of *D. saccharalis*.

The homologous competition assays demonstrated that Cry1Aa, Cry1Ac and Vip3Aa bound specifically to a protein in the *D. flavipennella* and *E. lignosellus* BBMV. In heterologous competition assays, Cry1Aa was able to compete Cry1Aa/Cry1Ac binding to this protein, so this likely represents a common Cry1A toxin binding protein in both insects, similar to Cry1A binding proteins of *H. zea*, *P. xylostella* and* P. gossypiella* (Karim et al., 2000; Estela, Escriche & Ferre, 2004; Gonzalez-Cabrera et al., 2006). However, Vip3Aa was unable to compete for Cry1Aa or Cry1Ac binding to this protein, so it represents a unique binding site for Cry1A toxins in these insects. Several authors have reported the absence of shared binding sites for Vip3Aa and Cry1Ac, Cry1Ab, Cry1Fa, Cry2Ae and Cry2Ab among different insects, as well as among Vip3Af with Cry1Ab and CryF for *S. frugiperda* (Lee, Miles & Chen, 2006; Sena, Hernandez-Rodrigues & Ferre, 2009; Liu et al., 2011; Gouffon et al., 2011; Ben Hamadou-Charfi et al., 2013; Chakroun & Ferré , 2014; Jakka, Ferré & Jurat-Fuentes, 2015).

**Figure 5:**
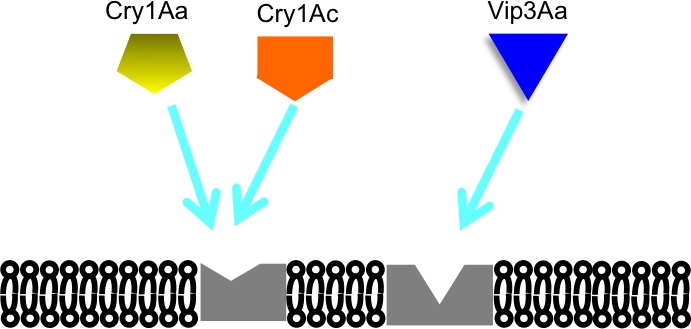
Proposed model of Cry proteins 1 and Vip3A in BBMVs from *D. flavipennella* and *E. lignosellus*.

Hence, after observing heterologous competitions among Cry1Aa, Cry1Ac and Vip3Aa, we could elaborate a binding model, as shown in Fig. 5 involving these toxins and binding proteins in the BBMV of *D. flavipennella* and *E. lignosellus*. However, Cry1 and Vip3 proteins can bind to more than one receptor. This model agrees with other data with competition between Cry1 towards a distinct binding site for Cry1 and Vip3A toxins in other insects (Sena, Hernandez-Rodrigues & Ferre, 2009; Sena, Hernandez-Rodrigues & Ferre, 2009; Chakroun & Ferré , 2014).

The heterologous competitions showed that Vip3Aa, when combined with Cry1Aa and Cry1Ac, do not compete for the same receptor, and in the ligand-blotting technique indicates more than one receptor for each protein. These two techniques should be interpreted separately, since in competition assays is possible only to verify if proteins share or not the same receptor. The fact that the protein can bind to several receptors present in the membrane does not contradict heterologous competition studies.

The toxin-binding model for Cry and Vip proposed for *D. flavipennella* and *E. lignosellus* argues against the combination of Cry1A genes in plants to be protected against these pests. Such combinations suggest that alleles may promote rapid resistance to more than one Cry1A toxin through alterations in the mutual binding site, similar to the model for Cry1A obtained in *H. armigera* (Estela, Escriche & Ferre, 2004). Likely, such changes in midgut binding sites will prove to be the most common means for insects to evolve field resistance against Bt insecticidal proteins.

Heterologous competition involving Cry1 toxins and *Heliothis* ssp BBMVs revealed three binding sites shared by Cry1Aa, Cry1Ab and Cry1Ac. Another site was found in competition assays with Cry1Ab and Cry1Ac. On the other hand, Cry2A and Vip3A seem to have only one binding site which is distinct from that found for Cry1A (Lee, Miles & Chen, 2006; Gouffon et al., 2011).

Several resistance management strategies have been proposed for Bt-transgenic crops, one of which is pyramided plants harboring more than one transgene. This strategy involves different toxins that use alternative sites on midgut receptors of the targeted insects.

This study stresses the importance of establishing binding models for Cry and Vip proteins as a primary tool to develop effective pyramided Bt-crops (Hernández-Rodríguez et al., 2013). We found that Cry1Ac/Vip3Aa have the potential to control *E. lignosellus*, and perhaps *D. flavipennella*, but more studies are needed. Data from these studies can be used to develop gene construct for sugarcane transgenic plants to control *D. flavipennella* and *E. lignosellus*.

## Conclusion

The data presented herein reveal the potential *B. thuringiensis* insecticidal action of Cry1Ac and Vip3Aa against *E. lignosellus* and perhaps *D. flavipennella*. Vip3Ca is not indicated for control of either pest, and Cry1A toxins should not be pyramided. Binding proteins were found for Cry1Aa, Cry1Ac, and Vip3Aa from BBMV of both insects. Binding assays suggested a model with two distinct receptors (Cry1Aa/Cry1Ac, and Vip3Aa). Among the studied proteins, the pair Cry1Ac/Vip3Aa hold the most promise for pyramided transgenic Bt sugarcane for control *D. flavipennella* and *E. lignosellus*. Future evaluation of synergism and antagonism of Cry1Ac and Vip3Aa is needed prior to development of transgenic sugarcane to control these pests.

##  Supplemental Information

10.7717/peerj.2866/supp-1 Supplemental Information 1 Raw data

## Additional Information and Declarations

### Data Availability

The following information was supplied regarding data availability: The raw data has been supplied as a Supplementary File.
